# Supplementary material for: Association between oral microbiome and seven types of cancers in East Asian population: a two-sample Mendelian randomization analysis
Source: Front Mol Biosci. 2023 Nov 21;10:1327893. doi: 10.3389/fmolb.2023.1327893 (PMC10702768; doi:10.3389/fmolb.2023.1327893)
Supplement: Supplementary file 1 [file DataSheet1.ZIP › Supplementary Materials/MR plots for tongue/tongue═╝/Pancreatic cancer/╥╚╧┘░⌐_╔╕╤í╜ß╣√/pheno.3013_to_pancreatic cancer_forest.pdf]

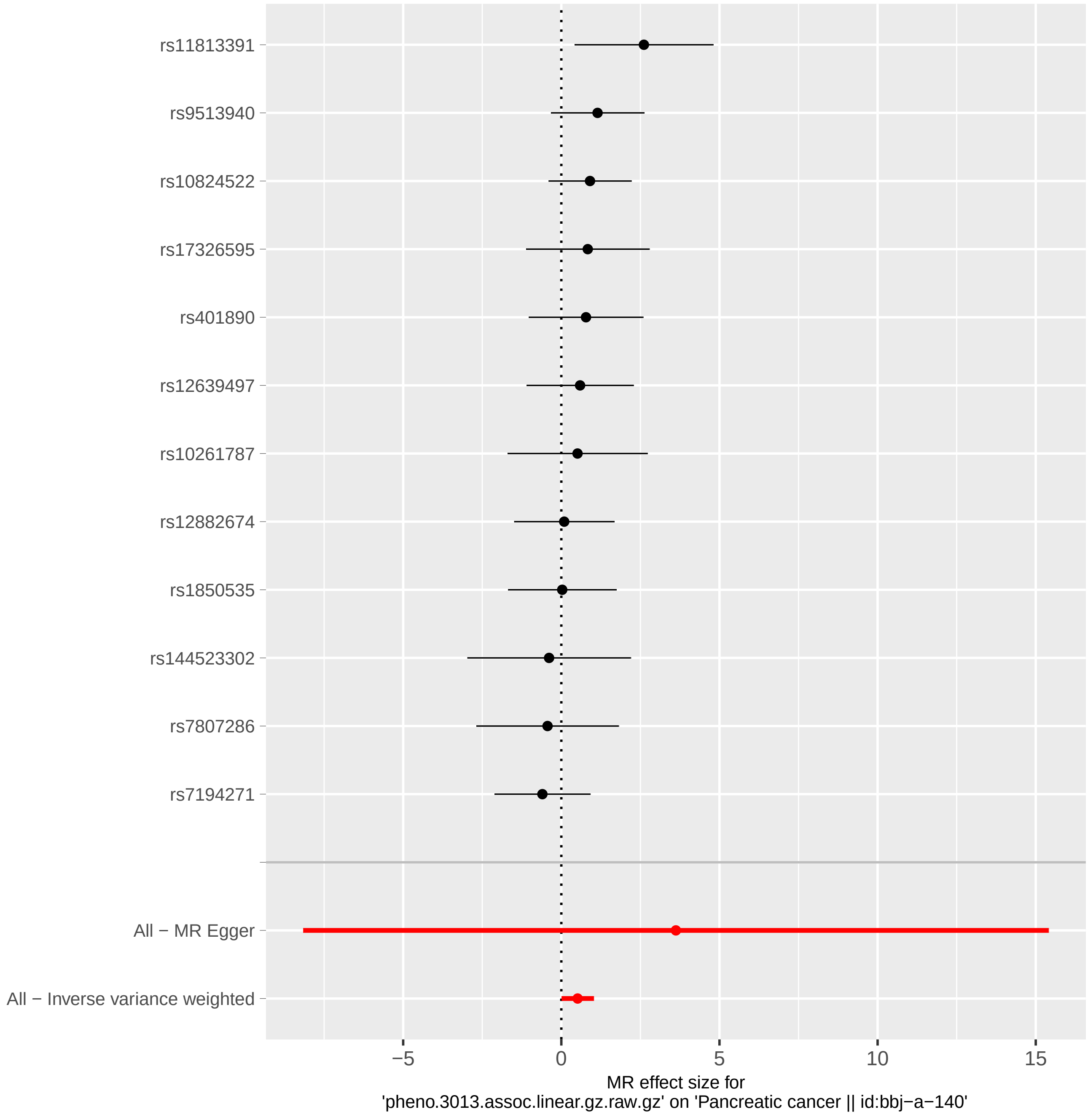
rs11813391
rs9513940
rs10824522
rs17326595
rs401890
rs12639497
rs10261787
rs12882674
rs1850535
rs144523302
rs7807286
rs7194271
All − MR Egger
All − Inverse variance weighted
−5
0
5
10
15
MR effect size for
'pheno.3013.assoc.linear.gz.raw.gz' on 'Pancreatic cancer || id:bbj−a−140'
